# Predictors of academic career progression among early career physician-scientists via an intensive research training program abroad: a case study

**DOI:** 10.1186/s12909-023-04069-8

**Published:** 2023-02-06

**Authors:** Shuang Liao, Christopher Lavender, Huiwen Zhai, Xinxi Zhou

**Affiliations:** 1 Department of Scientific Research and Education, Sun Yat-sen University Cancer Center, State Key Laboratory of Oncology in South China, Collaborative Innovation Center for Cancer Medicine, No. 651 Dongfeng East Road, Guangzhou, 510060 Guangdong, People’s Republic of China; 2 School of Sociology and Anthropology, Sun Yat-Sen University, Guangzhou , 510275 Guangdong, People’s Republic of China

**Keywords:** Physician-scientist, Academic career, Residency research training, Social Cognitive Career Theory, Self-efficacy

## Abstract

**Background:**

Despite extensive efforts to revitalize the physician-scientist pipeline, attrition has been observed along the physician-scientist developmental pathway. Research exposure during clinical training is considered an important factor favoring the decision to pursue an academic career pathway.

**Methods:**

The authors sought to identify factors associated with academic career progression among junior physician-scientists following the completion of an intensive research training program, using the framework of the Social Cognitive Career Theory (SCCT), to benefit the design of efforts to revitalize the physician-scientist career pipeline. We conducted a retrospective study of 108 physicians who completed a long-term research training program abroad during residency, or within a few years post-residency completion, between 2010 and 2017. With potential predictors of academic career progression prioritized by SCCT, multivariable logistic regression was used to identify predictors of sustained research involvement, high productivity and high research competency after training, respectively. The SCCT was used to illuminate our findings.

**Results:**

Co-publications with training supervisors abroad and medical oncology/pediatric oncology as a clinical specialty were positively associated with sustained research involvement and high productivity. Joining the training program after the age of 36 was negatively associated with high research competency. All of the predictors shared a common feature of high correlation with both self-efficacy and environmental elements, the reciprocal interactions of which may affect the career progression of physician-scientists.

**Conclusions:**

Insights gained through this analysis provide policy recommendations for the designing of efforts to revitalize the physician-scientist career pipeline. Priorities should be given to institutional oversight to ensure strengthened self-efficacy at the beginning of one’s academic career, by providing long-term research training opportunities to young residents and promoting co-publications with their training supervisors during the training. In order to avoid the negative impact to self-efficacy caused by patient-related burnout or academic isolation, academic medical centers should take measures to guarantee protected research time, and to develop a positive culture encouraging mentoring relationships between junior and experienced physician-scientists in medical departments.

## Introduction

With health profession training and a research track, physician-scientists gain insights into unmet clinical needs, and are at the forefront of endeavors in translating basic scientific discoveries into clinical applications [1]. Today, physician-scientists are driving advances in medical care with even more diverse endeavors, such as developing novel cellular therapies [2], gene therapies [3] and integrating artificial intelligence into medical practice [4]. Despite unprecedented opportunities for conducting basic and translational research in the biomedical sciences, there have long been concerns about a vanishing physician-scientist workforce [5–9]. A report from the National Institute of Health (NIH) Physician-Scientist Workforce (PSW) Working Group revealed disturbing trends of the PSW: The percentage of physicians devoted to research has fallen. Only 1.5% of the nearly 1 million MD physicians in the United States considered research as their main endeavor. Although it remained stable in size, the average age of the PSW is rising, due to an insufficient number of young physicians joining the workforce [9]. The reasons for this are multifaceted [8, 10–14]. Challenges include educational debt, training duration, increased clinical duties, difficulties in securing research funds, a lack of role models and mentors for junior researchers and a lack of research exposure during residency and fellowship.

In the face of this crisis, extensive efforts have been made to revitalize the physician-scientist career pipeline. Various research enrichment programs have been established, such as structured MD/PhD programs, the NIH Clinical Research Training Program for medical students [15–18], and residency research training programs [19]. Investigators have analyzed the factors that influence the career choice of MD and MD/PhD candidates [13], and the reasons behind the high attrition rates for medical school faculty [20–22]. Others have tried to elucidate the predictors of academic success among structured MD/PhD program graduates [23, 24]. Andriole and Jeffe found that attending schools with Medical Scientist Training Program funding and completing ≥ 1 year of research during residency were positively associated with full-time faculty appointments among US MD/PhD graduates. Skinnider et al., has also suggested that research productivity during MD-PhD training and the pursuit of additional research training during residency should be prioritized for MD-PhD programs.

To provide sufficient research exposure and to promote research competency among junior physician-scientists, 108 Sun Yat-sen University Cancer Center (SYSUCC) physicians during residency or within a few years post-residency completion, were sent abroad for intensive research training of around one year in biomedical laboratories at internationally renowned institutes, including academic medical centers and universities, between 2010 and 2017. Among the 108 trainees, 82 received their training in the United States, making it the most popular training destination. MD Anderson Cancer Center was the most popular institute, which hosted 23 young investigators over eight years.

**Fig. 1:**
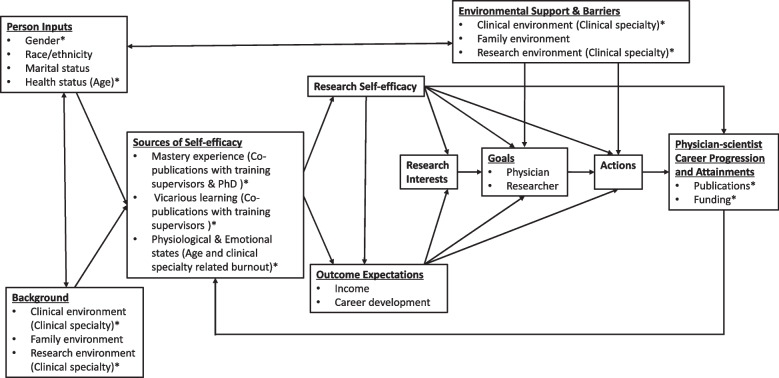
A depiction of the Social Cognitive Career Theory applied to physician-scientists’ academic career progression. Adapted from “Toward a unifying social cognitive theory of career and academic interest, choice and performance” by Lent et al. [26]. * Potential factors and metrics included in this study

Although the importance of long-term residency research training (≥ 1 year) is repeatedly emphasized in previous studies, the factors that may influence future academic career progression among the trainees still requires evaluation. In order to identify vital factors associated with long-term academic career progression among SYSUCC physicians following the completion of the training, which may benefit the design of future efforts to revitalize the physician-scientist career pipeline, we adopted the Social Cognitive Career Theory (SCCT) to help prioritize potential factors for regression analysis. Rooted in Bandura’s general social cognitive theory [25], the SCCT was proposed by Lent et al. in 1994 [26]. Its main area of concern is the interplay between various person, environmental and behavioral variables, which affect people’s academic and career interests, choices and performance outcomes (See Fig. 1). The SCCT asserts that self-efficacy is one of the vital factors that influences career choice and attainment, and it is constantly under the influence and reciprocal interactions of person factors and environmental elements. Self-efficacy perceptions are strengthened by mastery experiences (personal success), vicarious learning (exposure to role models), social persuasions and positive affective reactions [26]. In two former studies carried out by Bierer et al., and Lipira et al., the SCCT has been used as a “lens” for the evaluation of research curriculums and training programs at medical schools [27, 28]. Both studies observed an immediate increase in research self-efficacy after the training, while its correlation with the long-term research productivity, one of the main metrics evaluated in this study, remains elusive. The SCCT has also served as a framework in the identification of predictors for scientific productivity among academic staff at universities [29].

As the SCCT includes variables hypothesized to shape the career trajectories of physician-scientists [30], it was adopted in this study to prioritize potential factors for regression analysis and to illuminate our findings (See Fig. 1). Gender and age were included as factors of person inputs. As certain medical disciplines are believed to have historically provided a protected environment for the development of physician-scientists’ careers [31], and an association of clinical specialty with symptoms of burnout was also observed among US resident physicians [32], the trainee’s clinical specialty was included as a potential factor of both environmental elements and emotional states. Having a PhD was included as an indicator of mastery experience. In a previous study, prior publications have been proven to contribute to self-efficacy among university academic staff [29]. Therefore, we further included co-publications with training supervisors as an indicator of both mastery experience and vicarious learning (an effective mentoring relationship) during the training. Other factors, such as family environment and marital status, were excluded due to a lack of reliable data sources. Race/ethnicity was also excluded since no racial difference was found within the cohort.

In our study, publications and funding were included as metrics of physician-scientists career progression and attainments, as they were included as metrics of extrinsic career success in a comprehensive career-success model for physician-scientists [33]. The amount of SCI publications, receipt of national research funds and the impact factors (IFs) of academic journals have previously been documented as indicators of academic achievement or sustained research involvement of physician-scientists [31, 34, 35]. Although there are other important metrics indicating academic career progression, such as professional meeting presentations, tools/resources/assay development, expert panel participation and awards, they were not included in our study due to a lack of complete and reliable data sources.

In order to benefit the design of future efforts to revitalize the physician-scientist career pipeline, we further explored the logical associations between the identified factors and academic career progression of the trainees in light of the SCCT.

## Methods

### Background

SYSUCC mainly supports trainees during residency or within a few years post-residency completion to pursue basic or translational research training with up to one-year protected research time abroad. The inclusion criteria included a strong research background and a commitment to an academic career. Priorities are given to applicants with rich research experience, such as a PhD in biomedical sciences or having published original research articles as the lead author. Program applicants were required to identify a potential supervisor, and to draft a preliminary research plan together with him/her. Final approval depended on the general consideration of the quality of the research plan and the applicant’s research background. Support and coordination from the clinical department head was also important to guarantee better integration of the research training program into their clinical training and practice. Through mentored research, all the trainees gained precious training experience in the laboratories abroad. Progress of the research projects were communicated through weekly group meetings. Based on the needs of specific trainees, 1:1 mentorship meetings were irregularly scheduled for guidance and discussions regarding the projects and specific experiments. An extension of no more than three years was permitted for a few excellent scholars upon SYSUCC approval, to support the development of important scholarly output. Funding resources included SYSUCC/SYSU, the Postdoctoral Program of the Chinese Scholarship Council (CSC) and the Li Liqing Public Welfare Fund, established by a private donation to SYSUCC.

### Data collection

We considered the following three outcomes as positive indicators of sustained research involvement and high productivity: (i) receipt of National Natural Science Foundation of China (NSFC) funds since completing the training (an indicator of sustained research involvement); (ii) a high number of SCI publications since completing the training; and (iii) a high number of SCI publications as corresponding author since completing the training (indicators of high research productivity), and one outcome as a positive indicator of high research competency: publications with a high IF 1–6 years after the training.

Binary variables were created for the outcome variables for logistic regression analysis: (i) the number of SCI publications since completing the training program were categorized, with 12 or more considered high; (ii) the number of SCI publications with corresponding authorship since completing the training program were categorized, with eight or more considered high; (iii) the average IF 1–6 years after the training program was categorized, with four or more considered high.

We obtained information regarding funding from the NSFC, SCI publications and the IF of each journal from SYSUCC’s Office of Scientific Research Management for the 108 training participants enrolled between 2010 and 2017. This cohort was chosen based on the availability of the data, from 2007 to 2020. In order to analyze the factors which affected IF over the long-term, we created a subgroup of 58 trainees enrolled from 2010–2014. SCI publications were verified via PubMed for data accuracy. Tracked NSFC funds were the NSFC Youth Grant, and the NSFC General Grant. Publications were limited to original studies and reviews published in SCI journals with the trainees as the first/co-first or corresponding/co-corresponding author. The only exception was made for papers co-published with the research training supervisors abroad. There was no specific restriction on lead authorship.

As mentioned before, five variables were considered as potential factors which may affect participant’s academic career progression after the training based on the SCCT. Among them, gender and clinical specialty have previously been considered potential indicators of research involvement and faculty appointment among MD-PhD program graduates [23, 24].

Based on literature searches, we further created a three-category variable for clinical specialty, consisting of: (i) medical oncology and pediatric oncology; (ii) surgical specialties and radiation oncology; and (iii) specialties related to the Platform Departments (reference group), such as the Department of Pathology, Medical Imaging, Anesthesiology, Minimally Invasive Interventional Therapy, Endoscopy and Clinical Nutrition. A two-category variable, ≤ 36 and > 36, was created for age at the time of joining the training for the analysis of a high IF 1–6 years after the training.

### Statistical analysis

For the 108 physician-scientists enrolled in the training program from 2010–2017, we reported adjusted odds ratios (adjusted OR) and 95% confidence intervals (CI) from the multivariable logistic regression models to identify independent predictors of sustained research involvement and high productivity. To identify predictors of research competency over the long term, a 58 physician-scientist subgroup who were enrolled in the training from 2010–2014 was chosen for multivariable logistic regression analysis. In each case, we controlled for gender, since it is indicated as a potential factor related to research success [36]. The regression analysis for research involvement and productivity were further controlled for time since completing the training, because all the three binary dependent variables were created from continuous variables accumulating over time. The regression analysis for research competency was further controlled for clinical specialty, since differences in IFs have been observed among different subject areas in the Thomson Reuters Journal Citation Report [35]. Statistical analysis was performed using IBM SPSS Statistics, version 24 (IBM Corporation, 1989, 2016). A two-sided *P* value of < 0.05 was considered statistically significant.

## Results

### Characteristics of physician-scientists enrolled in the training program

Our sample included 108 physician-scientists from SYSUCC who participated in the training program from 2010 to 2017, with an average age mean ± standard deviation (SD) of 37.17 ± 4.81 at the time of joining the training. Among them, 89 (82%) had both MD and PhD degrees before the training, and 67 (62%) had co-publications with training supervisors abroad during or after the training. The majority of them showed high research involvement as well as productivity since completing the training: 59 (55%) were awarded at least one NSFC funding, 39 (36%) published more than 11 SCI publications as first or corresponding author, and 34 (31%) had more than seven SCI publications as corresponding author, within an average duration of 6.18 ± 2.22 years (mean ± SD, see Table 1). They also exhibited impressive research competency, based on the average IF of their publications after the training: 4.02 ± 2.54 (mean ± SD) for the whole group 1–3 years after, and 4.05 ± 1.37 (mean ± SD) for the subgroup 1–6 years after.

**Table 1:** Characteristics of physician-scientists at SYSUCC who participated in the research training program from 2010–2017 (*N* = 108)

Variables	
*Dependent variables: positive indicators of research career progression after the training*	
Receipt of NSFC Funds since completing the training	
Yes: No (%)	59:49 (54.6:45.4)
A high number of SCI publications since completing the training	
> 11: ≤ 11 (%)	39:69 (36.1:63.9)
A high number of SCI publications with corresponding authorship since completing the training	
> 7: ≤ 7 (%)	34:74 (31.5:68.5)
*Confounding variables*	
Gender	
Male: Female (%)	68:40 (63.0:37.0)
Time to training completion, years	
Mean ± SD	6.18 ± 2.22
*Independent variables*	
Receipt of a PhD degree before the training	
MD/PhD: MD/MSc (%)	89:19 (82.4:17.6)
Co-publications with training supervisors abroad	
Yes: No (%)	67:41 (62.0:38.0)
Clinical specialty	
Medical oncology and pediatric oncology (%)	22 (20.4)
Surgical specialties and radiation oncology (%)	49 (45.4)
Specialties related to the Platform Departments (%)	37 (34.3)
Age at the time of joining the training	
Mean ± SD	37.17 ± 4.81

*SYSUCC* Sun Yat-sen University Cancer Center

Platform Departments include the Department of Pathology, Medical Imaging, Anesthesiology, Minimally Invasive Interventional Therapy, Endoscopy and Clinical Nutrition

### Multivariable logistic regression

When adjusted for gender and time since completing the training, participants who co-published with training supervisors abroad had significantly greater odds of sustained research involvement and high productivity as indicated by all three binary outcomes (see Table 2). The trainees with a PhD degree before the training had significantly greater odds in securing NSFC funding since completing the training (adjusted odds ratio, 7.60; 95% CI, 1.54–37.43, *P* < 0.05), while specializing in medical oncology/pediatric oncology (adjusted odds ratio, 6.09; 95% CI, 1.33–28.01, *P* < 0.05), and being older at the time of joining the training (adjusted odds ratio, 1.20; 95% CI, 1.06–1.36, *P* < 0.01) was also positively associated with a higher number of SCI publications as the corresponding authors since completing the training. Trainees in surgical specialties/radiation oncology had significantly greater odds of high IF publications 1–6 years after the training (adjusted odds ratio, 17.09; 95% CI, 2.94–99.43, *P* < 0.01, see Table 3).  Conversely, when adjusted for gender and clinical specialty, being above 36 years old at the time of joining the training decreased the odds of having publications with high IFs 1–6 years after the training (adjusted odds ratio, 0.15; 95% CI, 0.04–0.61, *P* < 0.01).

**Table 2:** Logistic regression predicting academic career progression among training participants enrolled from 2010 to 2017 (*N* = 108)

	**Adjusted odds ratio (95% CI)**		
	**Receipt of NSFC Funds since completing the training**	**A high number of SCI publications since completing the training**	**A high number of SCI publications as corresponding author since completing the training**
Gender			
Male	0.72 (0.25–2.04)	1.63 (0.54–4.88)	0.95 (0.32–2.84)
Female	Reference	Reference	Reference
Age at the time of joining the training	0.93 (0.83–1.04)	1.03 (0.92–1.16)	**1.20 (1.06–1.36)**^**†**^
Time to training completion, years	**1.61 (1.24–2.09)**^**†**^	**1.87 (1.42–2.46)**^**†**^	**1.79 (1.35–2.38)**^**†**^
Co-publications with training supervisors abroad			
Yes	**4.75 (1.58–14.34)**^**†**^	**4.86 (1.53–15.47)**^**†**^	3.52 (1.16–10.72)*
No	Reference	Reference	Reference
Clinical specialty			
Medical oncology/pediatric oncology	2.33 (0.52–10.33)	3.25 (0.75–14.21)	6.09 (1.33–28.01)*
Surgical specialties/radiation oncology	0.99 (0.32–3.08)	1.68 (0.52–5.48)	2.01 (0.59–6.85)
Specialties related to the Platform Departments	Reference	Reference	Reference
Receipt of a PhD degree before the training			
Yes	7.60 (1.54–37.43)*	5.01 (0.81–31.01)	6.27 (0.83–47.36)
No	Reference	Reference	Reference

Values with statistical significance are in bold

*SYSUCC * Sun Yat-sen University Cancer Center, *NSFC * the National Natural Science Foundation of China, *CI*   Confidence interval

Platform Departments include the Department of Pathology, Medical Imaging, Anesthesiology, Minimally Invasive Interventional Therapy, Endoscopy and Clinical Nutrition

^*^ *P* < 0.05

^†^ *p* < 0.01

**Table 3:** Logistic regression predicting research competency among training participants enrolled from 2010 to 2014 (*N* = 58)

	**Adjusted odds ratio (95% CI)**
	**High impact factor (IF) 1–6 years after the training**
Gender	
Male	0.65 (0.16–2.71)
Female	Reference
Age at the time of joining the training	
> 36	**0.15 (0.04–0.61)**^**†**^
≤ 36	Reference
Co-publications with training supervisors abroad	
Yes	1.42 (0.38–5.25)
No	Reference
Clinical specialty	
Medical oncology/pediatric oncology	6.48 (0.87–48.30)
Surgical specialties/radiation oncology	**17.09 (2.94–99.43)**^**†**^
Specialties related to the Platform Departments	Reference
Receipt of a PhD degree before the training	
Yes	3.17 (0.22–45.20)
No	Reference

Values with statistical significance are in bold

*SYSUCC * Sun Yat-sen University Cancer Center, *CI*   Confidence interval

Platform Departments include the Department of Pathology, Medical Imaging, Anesthesiology, Minimally Invasive Interventional Therapy, Endoscopy and Clinical Nutrition

^†^ *p* < 0.01

## Discussion

There have long been concerns about a vanishing physician-scientist workforce. Despite extensive efforts to revitalize the physician-scientist career pipeline, considerable attrition has been observed along the physician-scientist developmental pathway. Research exposure during clinical training, among many other factors, have been considered crucial in favoring the decision to pursue an academic career pathway. The aim of this study was to define the predictors of academic career progression among early career physician-scientists after an intensive research training program, to benefit future programs alike, and to better inform their design strategies in revitalizing the physician-scientist pipeline.

There are several unique findings from our regression models, indicating new predictors of academic career progression. First, our analysis suggests that co-publications with training supervisors abroad is highly predictive of sustained research involvement and high research productivity after the training. And it is the only factor which is significantly associated with three of the four outcome variables. Mastery experiences (personal success) are considered one of the most reliable sources of self-efficacy in SCCT [37], thus reliable predictors of career decision and performance. As co-publications with training supervisors abroad reflects both personal success (source of self-efficacy) and an effective mentoring relationship (environmental support), the finding adds credibility to our use of the SCCT as an analytical framework. Self-efficacy has also served as a key predictor of performance among medical students in former studies [38, 39]. This finding emphasizes the role of universities and academic medical centers in promoting collaborative publications between the trainer and the trainee while designing training programs alike, which requires further institutional support such as sufficient funding for the training program, and integrated manuscript writing workshops.

In previous studies, being female was considered a negative factor for the academic career development of physician-scientists due to the latent gender bias, and the challenges of caregiving responsibilities [40, 41]. As having SCI publications 1–3 years before the training represents academic success of the trainees in the past, one may speculate that it may also be related to future academic career progression. However, no significant correlations were found between either of these two factors and any of the four outcome variables (data not shown for the latter) in our study cohort. We propose that strengthened self-efficacy after the training was the key for career success among its female participants, or those with no publications in the past, as individuals with high self-efficacy are more likely to persist regardless of temporary obstacles or past performance, and self-efficacy perceptions fluctuate and evolve in concert with mastery experiences/successful performances [42]. This is in line with the results of former studies which emphasize the role of mentoring in the career promotion of female physicians [33, 43]. Expectedly, in our study, having a PhD degree is only positively related to receipt of NSFC funds, since having a PhD degree may be considered an advantage during the subjective evaluation process. And age at the time of joining the training is positively related to a high number of SCI publications as a corresponding author, which is also unsurprising since the majority of the group leaders are senior physicians-scientists. Among all the person input related factors (gender, age and MD/PhD degree), age was the only factor which has significant and meaningful association with academic career progression. We propose that the intrinsic nature of “age” is the key: self-efficacy perceptions fluctuate and evolve in concert with mastery of experiences/successful performances, as well as, the ever-changing emotional and environmental influences brought about by increasing age.

Age is the only factor that is significantly associated with research competency in the regression model when controlled for gender and clinical specialty, indicating the optimum entry point of the training. Joining the training program above the age of 36 is negatively associated with a higher research competency in the future. Coincidentally, a recent study from Japan revealed that physician-scientists older than 37 years-old had higher patient-related burnout scores [44]. As emotional states are one of the sources of self-efficacy in the SCCT, increasing burnout may potentially impede research competency development. Considering the long duration of MD and PhD training, we suggest that priorities should be given to promising young residents while offering research training opportunities alike, providing continuity in research training which is beneficial in the context of rapid progress in basic and translational sciences. By strengthening self-efficacy, one-year intensive research training may also relieve the anxiety among junior physicians from exceedingly long work hours. Lastly, mentorship, career guidance, and role models are critical for professional identity development and the career success of young physician-scientists [45, 46]. We hypothesize that younger trainees might also have a better chance of developing a mentoring relationship in clinical environments after the training, indicating positive contextual influences in the SCCT. Policy makers should also take measures to reduce the considerable time required before entering residency in general, such as further integration in medical and research curricula in structured MD-PhD programs.

Our finding that physicians specializing in medical oncology/pediatric oncology are positively associated with sustained research involvement and high productivity is consistent with previous studies [23, 24, 31, 47]. Brass et al., suggest that these specialties are more likely to provide a protected environment for physician-scientists’ career development [31]. An association between clinical specialty and symptoms of burnout was also observed among US resident physicians [32]. Training in emergency medicine and general surgery were found to be associated with higher risks of burnout relative to training in internal medicine. Therefore, SYSUCC physicians with a clinical specialty of medical oncology/pediatric oncology might enjoy a better research environment and emotional state, which collectively explains their better academic productivity in our study cohort in the framework of the SCCT. The context within which a physician-scientist is working is composed of both a research and clinical environment, each having different and often conflicting expectations, and they reciprocally interact with person factors and can affect the career progression of physician-scientists [30]. As high demands of clinical service may become a career barrier and diminish the self-efficacy of physician-scientists, academic medical centers should take measures to guarantee protected research time, especially in surgical and platform departments which usually have higher clinical workloads and a potentially less attractive research environment. This finding also emphasizes the role of academic medical centers in developing a positive culture encouraging mentoring relationships between junior and experienced physician-scientists, which will strengthen self-efficacy through vicarious learning, and help to encourage them to pursue an academic career pathway.

### Limitations

As we only focused on directly quantifiable metrics such as grants and publications, further studies of more comprehensive factors, such as infrastructure, clinical responsibilities, motivations and creativity of the scholars, is warranted to reveal a clearer and complete description of physician-scientists career success related factors. Although this study was conducted at an academic cancer center with a relatively small group of highly-motivated young physicians, and the follow-up time relatively short, these data provide convincing evidence for the predictors of research career success after training. Our analysis of the multivariable logistic regression models with the same group of independent factors provides reliable associations between the selected factors and distinct aspects of academic career progression.

## Conclusions

Within the theoretical framework of the SCCT, predictors of long-term academic career progression were identified among junior physician-scientists after completing a long-term intensive research training program abroad. All of the predictors share a common feature of high correlation with both self-efficacy and environmental elements, the reciprocal interactions of which may affect the career progression of physician-scientists. Our observations may be of interest to universities, academic medical centers, agencies and organizations that implement or provide funding to residency research training programs. Priorities should be given to institutional oversight to ensure strengthened self-efficacy at the beginning of an academic career, by providing long-term research training opportunities to young residents and promoting co-publications with their training supervisors. Insights gained through this analysis provide policy recommendations for the designing of efforts to revitalize the physician-scientist career pipeline. In order to avoid the negative impact to self-efficacy caused by patient-related burnout or academic isolation, academic medical centers should take measures to guarantee protected research time, and to develop a positive culture encouraging mentoring relationships between junior and experienced physician-scientists in medical departments.

## Data Availability

The datasets generated and/or analyzed during this current study are not publicly available due to privacy issues but are available from the corresponding author upon reasonable request.

## Abbreviations

SCCT: Social Cognitive Career Theory
SYSUCC: Sun Yat-sen University Cancer Center
CSC: Chinese Scholarship Council
NSFC: National Natural Science Foundation of China
